# Single Pass Laser Process for Super-Hydrophobic Flexible Surfaces with Micro/Nano Hierarchical Structures

**DOI:** 10.3390/ma11071226

**Published:** 2018-07-17

**Authors:** Hyuk-Jun Kwon, Junyeob Yeo, Jae Eun Jang, Costas P. Grigoropoulos, Jae-Hyuck Yoo

**Affiliations:** 1 Department of Information and Communication Engineering, DGIST, Daegu 42988, Korea; hj.kwon@dgist.ac.kr (H.-J.K.); jang1@dgist.ac.kr (J.E.J.); 2 Department of Physics, Kyungpook National University, Daegu 41566, Korea; junyeob@knu.ac.kr; 3 Department of Mechanical Engineering, University of California, Berkeley, CA 94720, USA; 4 Physical and Life Sciences and NIF and Photon Sciences, Lawrence Livermore National Laboratory, Livermore, CA 94550, USA

**Keywords:** super-hydrophobic, laser process, polytetrafluoroethylene (PTFE), polydimethylsiloxane (PDMS), hierarchical structures

## Abstract

Wetting has been studied in various fields: chemical industry, automobile manufacturing, food companies, and even life sciences. In these studies, super-hydrophobic surfaces have been achieved through complex steps and processes. To realize super-hydrophobicity, however, we demonstrated a simple and single pass laser process for the fabrication of micro/nano hierarchical structures on the flexible polytetrafluoroethylene (PTFE, Teflon) surface. The fabricated hierarchical structures helped increase the hydrophobicity by augmenting the surface roughness and promoting air-trapping. In addition, we employed a low-cost and high-throughput replication process producing numerous polydimethylsiloxane (PDMS) replicas from the laser-processed PTFE film. Thanks to the anti-adhesive characteristics of PTFE and the elasticity of PDMS, the structure perfectly transferred to the replica without any mechanical failure. Moreover, our designed mesh patterns offered the possibility of large area applications through varying the process parameters (pitch, beam spot size, laser fluence, and scan speed). Even though mesh patterns had relatively large pitch (190 μm), we were able to achieve high contact angle (>150°). Through pneumatically deformed structure, we clearly showed that the shape of the droplets on our laser-processed super-hydrophobic surface was spherical. Based on these outcomes, we can expect our single laser pulse exposure process can overcome many drawbacks and offer opportunities for advancing applications of the wetting phenomena.

## 1. Introduction

One can observe water droplets rolling off the surface of lotus leaves during rain. This hydrophobicity typically comes from the roughness of tiny micro-scale structures and water repellent materials. Depending on the surface condition (e.g., material and roughness) and tilt angle, droplets on the surface experience different accelerations as they are subject to gravity and/or airflow drag. Research on wettability of a solid surface and the water contact angle has been widely explored in industry and in academia through the years. The models of Wenzel (where the droplet is completely wet between microstructures) and Cassie (that assumes air is trapped between these micro- structures while the droplet stays on top of the microstructures and trapped air) are usually employed for describing the relation between the surface structure and hydrophobicity. To improve the lotus effect (i.e., reduce friction resistance and adhesion force), artificial super-hydrophobic surfaces characterized by water contact angle larger than 150° have attracted great attention in fundamental research and practical applications [1,2,3]. Various approaches have been reported by controlling the chemical composition [4,5,6] by fabricating micro/nano structures [7,8], modifying the physical morphology of the surface [9], and using water repellent materials [1,2,3,7]. Through the control of these surface characteristics, various useful practical applications are demonstrated, such as self-cleaning [10,11,12], anti-icing [13,14], water harvesting [15], no loss transport [16,17], and electrowetting [18,19]. However, due to technical issues, implementations have been limited. For example, to achieve the super-hydrophobic surface, complicated multiple steps are typically required. Also, these techniques cannot easily form hierarchical micro/nano structures promoting hydrophobicity via increasing the multiscale surface roughness. In this study, we directly wrote super-hydrophobic surfaces with nano and micro hierarchical structures on PTFE (McMaster-Carr, Elmhurst, IL, USA, Adhesive-Backed film) by single femtosecond (fs) laser pulse irradiation. Femtosecond laser (particularly single pass process) is used for industrial applications: mechanical testing [20] and tuning optical properties [21].

Super-hydrophobic process producing numerous polydimethylsiloxane (PDMS) (Sylgard 184, Dow Corning, Midland, MI, USA) surface was achieved by replication. Hierarchical polymer surfaces could be duplicated many times. In addition, we designed a specific mesh pattern for the scaling up of the effective area through parametric study. The results will be helpful for using pulsed laser processing to modify the wettability of the surface and for understanding the interaction between various micro/nano hierarchical structures and the water droplets. Also, we demonstrated droplet position control on pneumatically modified flexible super-hydrophobic surface where droplets easily move at a small tilt angle.

## 2. Result and Discussion

**Figure 1:**
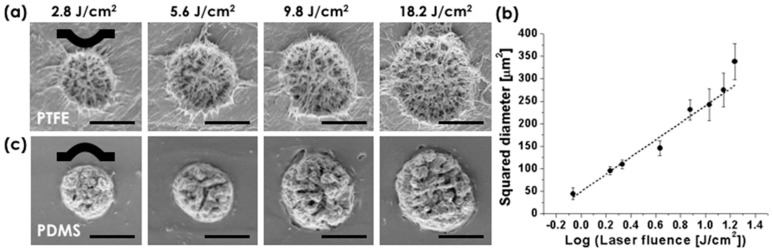
Single laser pulse ablation of polytetrafluoroethylene (PTFE) and process producing numerous polydimethylsiloxane (PDMS) process producing numerous polydimethylsiloxane (PDMS) replica: (**a**) scanning electron microscopy (SEM) pictures of ablated PTFE surface under varied laser fluence; (**b**) The squared diameter (*D*^2^) of the ablated PTFE spot is plotted as a function of the laser fluence in log scale; (**c**) SEM pictures of PDMS replica from the ablated PTFE surface (**a**). The scale bars are 10 μm.

Figure 1a shows scanning electron microscopy (SEM) images of the PTFE surface upon single laser pulse irradiation at varied laser fluences. Here, we note that PTFE offers very attractive physical and chemical properties: low friction resistance, low surface adhesion, thermal and chemical stability, biocompatible, and high water repellency [5,6]. These physical and chemical features allow formation of fiber-like micro/nano hierarchical structures unlike any other polymers (e.g., polyimide). This is unique phenomenon to PTFE offering a great advantage for easily generating super-hydrophobic surface through just a single femtosecond (fs) laser shot. Furthermore, all laser processes in this study were carried out with laser (Spitfire, Spectra-physics, Santa Clara, CA, USA) of ~100 femtosecond pulse duration, 400 nm wavelength, and 1 kHz repetition rate. The laser system was equipped with the XY translation stage (ANT130, Aerotech Inc., Clovis, NM, USA), a laser power attenuator (consisting of a half wave plate and a polarizing beam splitter), a mechanical shutter (Thorlabs, Newton, NJ, USA, SC10), and in-situ monitoring system. For focusing the laser beam, long working distance objective lenses (Mitutoyo, Takatsu ku, Japan) were used. In Figure 1a, ablative removal of PTFE material was observed in the center, resulting in a concave surface profile as presented with the black line. In addition, fibrous debris (i.e., the explosive ejection of molten PTFE material) was radially distributed along the perimeter during the laser ablation process [20]. Since PTFE exhibits its first linear absorption peak at 160 nm and the wavelength of the laser used in this study is 400 nm with 100 femtosecond (fs) pulse duration, laser energy should be absorbed by multi-photon process [22,23]. Figure 1b shows the squared diameter *D*^2^ of the ablated areas that is linearly proportional to the logarithm of the incident laser fluence. With *1/e*^2^ laser beam spot radius (w*_o_* = 6.44 μm), the ablation threshold (*Φ_th_*) is approximately calculated as 0.77 J/cm^2^ based on *D*^2^ *= 2w_o_*^2^ *ln(Φ_o_/Φ_th_*), where *Φ_o_* is the average laser fluence that is defined as the pulse energy (*E*) per irradiated area, *Φ_o_ = E/(πw_o_*^2^) [24]. In a low-cost and high-throughput process, this fast laser direct writing (LDW) is used as the master mold fabrication. Furthermore, laser assisted PTFE master molds with micro/nano hierarchical structures have been utilized to generate replicas on the surface of PDMS. Figure 1c shows SEM pictures of PDMS replicas from the ablated PTFE film, having convex surface profile as illustrated by the black line.

**Figure 2:**
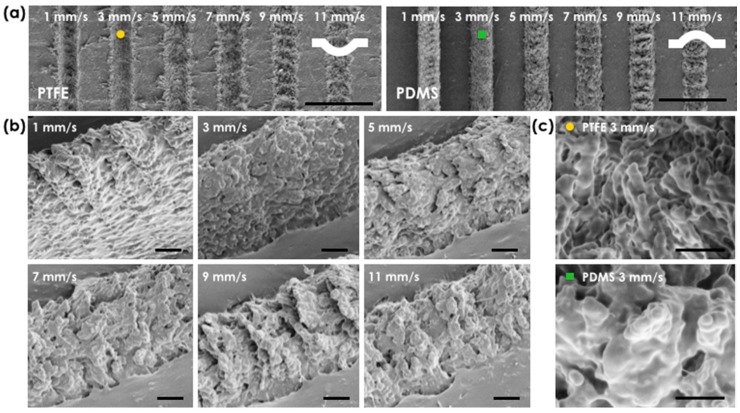
Line ablation with rough surface on a PTFE film by sample scanning: (**a**) SEM pictures of ablated PTFE film at the laser fluence of 56 J/cm^2^ and varied sample scanning speeds from 1 to 11 mm/s, and PDMS replica from the PTFE film. The scale bars are 50 μm. The white curved lines illustrate the surface profiles of PTFE and PDMS films; (**b**) Tilted SEM pictures of the PDMS replica. The scanning speed affects the height of PDMS replica (or the depth of PTFE template). The scale bars are 5 μm; (**c**) Magnified SEM pictures of rough PTFE and PDMS region (marking with circle and box in (**a**)) at the scanning speed of 3 mm/s. The scale bars are 2 μm.

We further investigate a scanning scheme for writing line patterns with micro/nano hierarchical surface. Upon laser irradiation, the motorized stage was translated at speed from 1 to 11 mm/s; the scanning speed of the laser was determined by the overlap between the laser pulses and the respective incident energy density distribution along the ablation line (Figure 2). Note that the repetition rate of the laser was set to 1 kHz. Figure 2a shows SEM images of a PTFE surface at the laser fluence of 56 J/cm^2^ and the corresponding PDMS replica image. The ablation line width did not depend much on the scanning speed, in contrast to the variation of processed depth. The depth of the ablated concave PTFE structures was directly reflected to the height of convex PDMS replicas as shown in tilted SEM pictures (Figure 2b). Height increase of the PDMS replica was observed as the scanning speed decreased. For example, at the scanning speed of 11 mm/s (maximum scan speed in our experiment), the pitch between adjacent laser pulses was 11 μm, and a train of adjacent single pulse ablation features was observed. On the other hand, at lower scanning speeds these distinctive periodic features could not be seen, and the height of PDMS replicas (or the depth of PTFE template) increased (or deepened) due to the pulse accumulation effect [20]. Therefore, a dramatic height increase was seen at our minimum scanning speed of 1 mm/s (or overlapped laser pulses with 1 μm pitch). Table 1 summarizes our findings for averaged height of PDMS replica under different laser fluences and scanning speeds of laser. When we magnify laser processed PTFE and PDMS region (marked with circle and box patterns in Figure 2a), the surface of laser ablated micron structure is decorated with nano roughness, resulting in micro/nano hierarchical structures. The magnified SEM pictures confirm that the pulsed laser process allows generation of complex micro/nano hierarchical structures without expensive, complicated, and multi-step processes involving vacuum and mask tools. Because the adhesion strength of the interface between PTFE and PDMS is relatively very low, additional chemical or physical anti-adhesive surface agent treatment is not necessary either; in order to produce a PDMS replica from a surface modified template, anti-adhesive coating is typically required [25,26].

**Figure 3:**
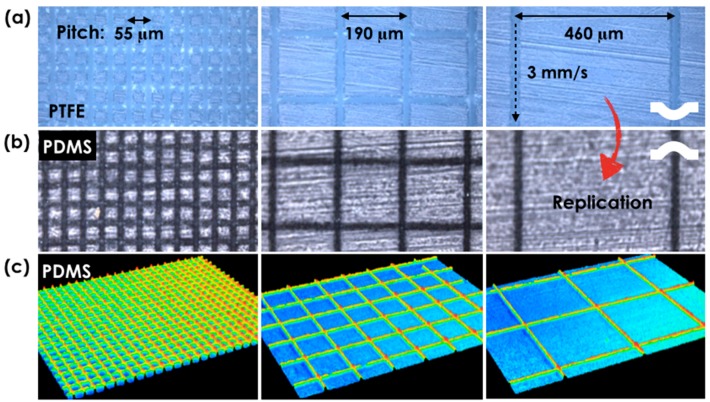
Direct PTFE surface morphology modification and PDMS replica: Microscopic pictures of (**a**) PTFE surface and (**b**) PDMS replica with varied pitches of 55, 190, and 460 μm, at the scanning speed of 3 mm/s. The white schematics describe the surface morphology; (**c**) Confocal microscopic pictures of PDMS replica with varied pitches of 55, 190, and 460 μm, at the scanning speed of 1 mm/s.

In order to control hydrophobicity over a large surface area, we designed mesh patterns of hierarchically structured lines with two design control parameters: pitch (55, 85, 130, 190, 265, 355, and 460 μm) and scanning speed (1, 3, 5, 7, 9, and 11 mm/s). Figure 3a shows three representative microscopic images of PTFE surfaces having different pitch of 55 (minimum), 190 (medium), and 460 μm (maximum) at the fixed scanning speed of 3 mm/s. In addition, PDMS patterns of the same design parameters were directly replicated from the PTFE surface as shown in the optical microscope images of Figure 3b. Confocal microscopic images of PDMS surface (Figure 3c), showed faithfully transferred convex mesh patterns.

**Figure 4:**
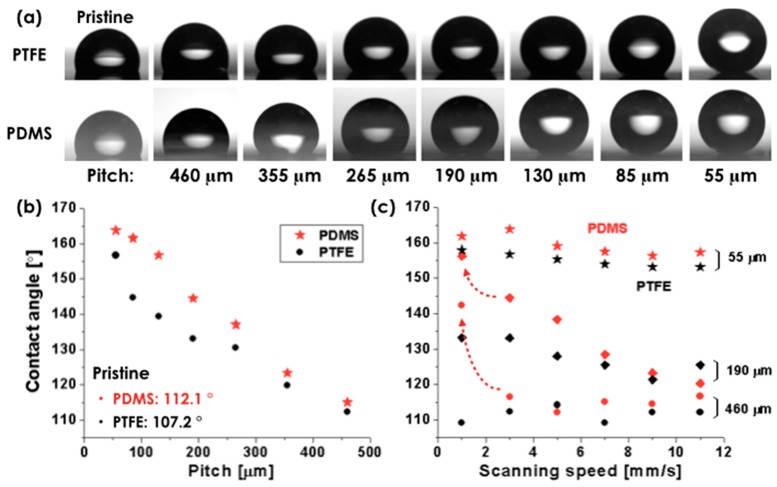
(**a**) Microscopic pictures of water droplet on surface modified PTFE sample and PDMS replica at the laser fluence of 35.0 J/cm^2^ and the scanning speed of 3 mm/s; (**b**) measured contact angle of the droplets in (**a**); and (**c**) contact angle of droplets on surface modified PTFE sample at varied scanning speeds and PDMS replica.

Contact angles of water droplets were measured on the laser-processed PTFE film with the designed patterns as well as on the PDMS replicas (described in Figure 3) and are presented in Figure 4. For the measurement, water droplets with the volume of ~1 μL were loaded on both the modified surfaces of PTFE film and the PDMS replica. Since our surfaces are rough with hierarchical structure, the wetting trend can be interpreted by the modified Young’s equations, either in the Wenzel model state (*cosθ′ = r (γ_sv_ − γ_sl_)/γ_lv_*, where *θ′* is contact angle, *r* is the roughness factor, and *γ_sv_*, *γ_sl_*, and *γ_lv_*, respectively denote the interfacial free energies per unit area of the solid-vapor, solid-liquid, and liquid-vapor interfaces) or in a Cassie–Baxter state (*cosθ′ = f cosθ + f − 1*, where *f* is an area fraction of wetted solid surface). We should note that Wenzel state (water droplet fills in the cavities or grooves on the surface) is dominant when the surface has small surface roughness, and Cassie’s model (air intrudes into the cavities on the surface and make air-water droplet interface) becomes effective as surface roughness increases. The models imply that the parameters *r* or *f* (i.e., increasing density of hierarchical structure) should increase in order to obtain high contact angle. Figure 4a,b show the side view of droplets placed on the modified surfaces with varied pitches (at the constant laser fluence of 35.0 J/cm^2^ and the fixed scanning speed of 3 mm/s) and their contact angles, respectively; the droplet becomes more spherical (i.e., increasing contact angle) as the line pattern pitch is decreased. These results are generally in line with our earlier expectations. However, a closer look revealed that wetting and contact angle exhibited some differences on surface with different surface free energy (PTFE: 18~26 mJ/m^2^ and PDMS: 15~22 mJ/m^2^) even though surfaces had the same geometry; the contact angle of pristine PTFE and PDMS surface was measured as 107.2° and 112.1°, respectively, because high free energy surfaces were more easily wet (i.e., low contact angle) than low free energy surfaces. In this respect, super-hydrophobic PDMS replica surface with a relatively low surface free energy compared to PTFE was achieved, when the pitch was 130, 85, and 55 μm. In contrast, super-hydrophobicity was achieved on PTFE surfaces only when the pitch was 55 μm. We also measured the contact angles dependence on the scanning speed for the representative three pitches (55, 190, and 460 μm) as shown in Figure 4c. The result shows that the contact angle of PTFE and PDMS replica increases as the scan speed decreases. An abrupt increase in the contact angle of PDMS replicas (marked by red dashed arrows in Figure 4c) was observed at the scanning speed of 1 mm/s, where a dramatic height increase of the hierarchical structure was obtained (see Table 1 in detail). This is because the PDMS replica sample has a convex surface; in contrast, the surface morphology of PTFE sample is concave. In the case of PDMS replica, convex protrusions on the surface can induce air pockets and their micro/nano hierarchical structures also can effectively promote the formation of air-trapping regime under the liquid. Moreover, we observed and discussed the large height increase at the scanning speed of 1 mm/s (see Figure 2 and Table 1). Therefore, the hydrophobic nature of the surface is further reinforced. On the contrary, in the case of PTFE sample, the micro/nano hierarchical structures cannot be beneficial for the hydrophobicity because the trapped air inside the concavely formed structures stops a drop from going to a place where there are many micro/nano hierarchical structures; a drop just rests on a surface without hierarchical structure, where the interfaces are all planar (composed of solid and air). Therefore, the contact angle was not highly affected by the scanning speed. Moreover, at the small pitch of 55 μm, the droplets could rest on top of the convex pillars only. At this point, the surface roughness no longer offered advantages with regard to promoting hydrophobicity; there were not much change of contact angles caused by the scan speeds at the pitch of 55 μm. These results show that we can make large effective super-hydrophobic surface with relatively big pitch (190 μm) that suits large-scale processing and related applications.

**Figure 5:**
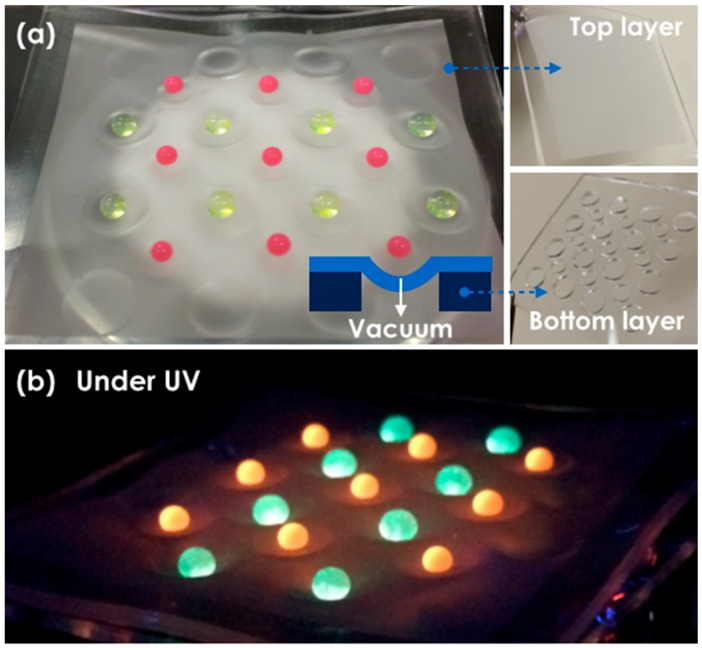
Pneumatically deformed flexible PDMS superhydrophobic film: (**a**) droplets with fluorescence particles were positioned on the deformed zone. The volumes of the red and yellow droplets were 5 and 10 μL, respectively; (**b**) a tilted photograph under UV irradiation.

Due to the low adhesion of the water droplet on our super-hydrophobic surface, it is rather difficult to localize a droplet on a specific surface spot by contact using a syringe needle or a micropipette. To overcome this practical issue, we utilized the flexibility of the super-hydrophobic PDMS replica film. To control deformation, we attached the super-hydrophobic PDMS top layer (5 cm × 5.5 cm) on the pristine PDMS bottom layer with through holes as shown in Figure 5a. Then, a glass vacuum filter holder (Whatman, Maidstone, UK, 1960-004) with 47 mm diameter was firstly combined on a neck of an opened side arm flask which was connected to a vacuum pump (FJC, Mooresville, NC, USA, 6912). As described in the schematics, the top layer over the hole was pneumatically deformed via a vacuum filtration apparatus. When droplets were dropped on the surface, they self-assembled in the center of the deformed zone (Figure 5a). We note that fluorescence particles (red and yellow) were used to improve the clarity of the droplets and ultraviolet (UV) light helped them to emit their distinct colors. In Figure 5b, we can clearly observe the deformed surface and the site-specifically located droplets with fluorescence emission under UV irradiation. Because the single pass laser process produced super-hydrophobic surface with micro/nano hierarchical structures, the shape of the droplets on the surface was approximately spherical.

## 3. Conclusions

In summary, we demonstrated a simple, single pass laser direct writing process for fabricating super-hydrophobic surface without requiring any chemical treatment and additional processes. Femtosecond laser irradiated PTFE film, incurring, absorption via a nonlinear multi-photon process. After the single laser scanning pass (or single shot for a stationary sample), micro/nano hierarchical structures were easily formed on the processed PTFE film. Also, super-hydrophobic PDMS replication was directly achieved from the laser processed PTFE template without requiring anti-adhesive coating treatment because of the anti-adhesive property (i.e., low surface free energy) of PTFE. Moreover, the process can be programmed with designed mesh patterns suitable for a large area. Therefore, we expect the process offers time and cost reduction to form super-hydrophobic flexible surfaces and may emerge as a promising technology.

## Figures and Tables

**Table 1:** Averaged height (μm) of PDMS replica (or averaged depth of PTFE surface) under varied laser fluences and sample scanning speeds.

Laser Fluence (J/cm^2^)	Scanning Speeds (mm/s)					
	Pitches between Pulses (μm)					
	1	3	5	7	9	11
24.5	24.3	12.6	7.2	6.5	4.4	3.0
35.0	32.0	14.0	8.2	7.0	5.5	4.0
45.5	37.6	17.1	10.1	8.0	6.8	5.7
56.0	40.3	18.0	11.5	8.6	7.3	6.2
